# Using relative survival measures for cross-sectional and longitudinal benchmarks of countries, states, and districts: the BenchRelSurv- and BenchRelSurvPlot-macros

**DOI:** 10.1186/1471-2458-13-34

**Published:** 2013-01-14

**Authors:** Christian O Jacke, Iris Reinhard, Ute S Albert

**Affiliations:** 1 Central Institute of Mental Health, Medical Faculty Mannheim/University Heidelberg, Square J5, 68159 Mannheim, Germany; 2 Department of Gynecology, Gynecological Endocrinology and Oncology, Breast Center Regio, University of Marburg, Marburg, Germany

**Keywords:** Benchmark*, Prevention & control, Outcome assessment, Relative survival, Registries, Breast cancer

## Abstract

**Background:**

The objective of screening programs is to discover life threatening diseases in as many patients as early as possible and to increase the chance of survival. To be able to compare aspects of health care quality, methods are needed for benchmarking that allow comparisons on various health care levels (regional, national, and international).

**Objectives:**

Applications and extensions of algorithms can be used to link the information on disease phases with relative survival rates and to consolidate them in composite measures. The application of the developed SAS-macros will give results for benchmarking of health care quality. Data examples for breast cancer care are given.

**Methods:**

A reference scale (expected, E) must be defined at a time point at which all benchmark objects (observed, O) are measured. All indices are defined as O/E, whereby the extended standardized screening-index (eSSI), the standardized case-mix-index (SCI), the work-up-index (SWI), and the treatment-index (STI) address different health care aspects. The composite measures called overall-performance evaluation (OPE) and relative overall performance indices (ROPI) link the individual indices differently for cross-sectional or longitudinal analyses.

**Results:**

Algorithms allow a time point and a time interval associated comparison of the benchmark objects in the indices eSSI, SCI, SWI, STI, OPE, and ROPI. Comparisons between countries, states and districts are possible. Exemplarily comparisons between two countries are made. The success of early detection and screening programs as well as clinical health care quality for breast cancer can be demonstrated while the population’s background mortality is concerned.

**Conclusions:**

If external quality assurance programs and benchmark objects are based on population-based and corresponding demographic data, information of disease phase and relative survival rates can be combined to indices which offer approaches for comparative analyses between benchmark objects. Conclusions on screening programs and health care quality are possible. The macros can be transferred to other diseases if a disease-specific phase scale of prognostic value (e.g. stage) exists.

## Background

In many diseases early detection and secondary prevention are of central importance [1-3]. Life-threatening malignant diseases are therefore divided into stages according to their disease phase. They are recorded using international nomenclatures (e.g. Union for International Cancer Control, American Joint Committee in Cancer) [4,5]. Thus, screening programs are aimed at discovering diseases in as many patients and as early as possible to increase their chance of survival.

First approaches on how the number of patients per disease phase/stage can be linked to the corresponding chance of survival are based on Beatty et al. [6,7]. They developed a series of benchmark-algorithms that address different aspects of health care. Beatty and colleagues described a screening index based upon the sum of the products of the stage number (0–4) and the number of cases at that stage divided by the total number of cases. Using the stage number in the calculation was considered arbitrary and it was replaced by the national 5-year mortality for that stage termed the case-mix index for the institution or region. This was then standardized by comparison to the national case-mix index and termed the standardized case-mix index (SCI). They also described a standardized work-up index (SWI) to address the issue of ‘upstaging’ of cases and a standardized treatment index (STI) to evaluate outcome using institutional or regional mortality (stage and overall) compared to national mortalities. The product of the SCI, SWI and STI defined the composite measure named overall performance evaluation (OPE). They used these indices and evaluations to compare different regions over the same time interval (cross-sectional analysis perspective) and the same institution across different time intervals (longitudinal analysis perspective).

However, the screening-index was not standardized, reflected a negative association with the observation period, and, thus, was not included in the OPE. In addition, the OPE had a logical weakness, because the standardized case-mix index was present in the denominator as well as the numerator and cancelled itself out. Finally, the approach was based on disease-specific cause of death statistics which required the modeling of competing risks according to the method of Gooley [8]. This method focuses on the mortality of one disease only and, therefore reduces the case numbers. This is of special importance for small geographic units that already have small case numbers as it is. This outcome-indicator also does not include background-mortality.

### Objectives

This contribution intends to overcome the existing shortcomings of the proposed algorithms and to extend the indices (see methods). Furthermore, the automation of the benchmark-algorithms (SAS-macros) will standardize the screening-index, produce a positive association with the observation time after appropriate mathematical conversion, and will adequately assess a composite measure. Disease-specific fatality rates will be replaced by relative survival rates as outcome-indicators. The macro results will be demonstrated by exemplary applications. The development of the indices and the main working hypothesis is built on the assumption that comprehensive early detection programs are capable of detecting diseases early, leading to disease stage shifts and facilitates clinical work-up which is associated with increasing relative 5-year survival rates. Improvements of health care quality should be illustrated transparently.

## Implementations

### Data

In general, population based databases with demographic information such as registers (e.g. cancer register) may be used. Clinical registers, cohort studies, or health care network databases are also suited if a high data quality and epidemiologic relevance is ensured [9-11]. The application example is based on the assumption that data of the Surveillance Epidemiology and End Results (SEER) [12] and the Norwegian cancer register [13] fulfill these requirements.

### Variables

The variables for the “best-performer“, that must be used to compare all others, define the reference object. All other comparative objects are designated as benchmark objects and must have identification numbers that can definitely be distinguished from one another. In addition, information on the year of first definite diagnosis (incidence year) and the disease phase (e.g. for cancer stage 0-IV) is necessary. The absolute and relative distribution of persons per disease phase (e.g. per stage) as well as the corresponding relative survival rates (e.g. 5 years) are needed. In addition, the total number of ill patients per time unit (e.g. year) and the relative overall survival are important. Table 1 serves as an example what specifically the SAS-Macro *BenchRelSurv* expects as a variable-set in the so-called long-format of SAS.

**Table 1:** Configuration of Data and Variable Sets

**Group**	**Year**	**Identification**	**Stage**	**Number per Stage**	**Number Total**	**Relative Survival**
**RefGroup**	**RefYear**	**RefID**	**RefStage**	**RefNpStage**	**RefN**	**RefRelSurv**
SEER17	1999-2003	1	0	167	191771	93.6
SEER17	1999-2003	1	1	83081	191771	100.00
SEER17	1999-2003	1	2	72195	191771	90.2
SEER17	1999-2003	1	3	12617	191771	61.3
SEER17	1999-2003	1	4	8449	191771	22.5
SEER17	1999-2003	1	5	15262	191771	77.3
SEER17	1999-2003	1	6	191771	191771	89.1

Legend: (Prefix Ref) Definition for reference object substitutable by prefix 'Bench' indicating benchmark object, (Stage) Stage of disease where 5='not known' and 6='total' overall respectively.

Source: New malignant breast cancer cases from Surveillance, Epidemiology, and End Results (SEER) Program (http://www.seer.cancer.gov) SEER*Stat.

Database: Incidence - SEER 17-Nov 2010 Sub (1973–2008 varying).

**Table 2:** Cross-Sectional Benchmarking of SEER17-Registers (1999–2003)

	**eSSI**	**SCI**	**SWI**	**STI**	**OPE**	**ROPI**
Reference Object (1999–2003)						
SEER 17	1.000	1.000	1.000	1.000	1.000	1.000
Benchmark Objects						
Iowa	0.951	0.993	1.032	1.023	1.048	1.110
New Mexico	0.973	0.959	1.023	1.037	1.017	1.090
Seattle (Puget Sound)	0.960	1.035	1.033	1.004	1.074	1.080
New Jersey	0.991	0.938	0.997	1.052	0.983	1.059
Utah	0.991	0.987	1.026	1.010	1.022	1.045
San Francisco-Oakland (SF)	1.002	1.036	1.039	0.987	1.062	1.023
San Jose-Monterey (SJM)	0.983	1.049	1.009	0.991	1.049	1.018
Conneticut	0.973	1.020	0.985	1.000	1.005	1.013
California excl. SF/SJM/LA	0.994	1.018	1.006	0.990	1.014	1.002
Alaska Natives	0.981	0.932	0.956	0.980	0.874	0.955
Atlanta	1.024	1.001	0.982	0.982	0.965	0.941
Los Angeles (LA)	1.051	1.018	1.008	0.978	1.003	0.938
Kentucky	0.965	0.927	0.862	1.045	0.834	0.933
Detroit (Metropolitan)	1.029	0.981	0.935	0.995	0.913	0.905
Hawaii	0.975	1.078	0.926	0.948	0.947	0.900
Lousiana	1.074	0.960	0.952	0.975	0.891	0.865
Rural Georgia	0.947	0.884	0.709	1.076	0.674	0.805

Legend: (eSSI) Extended Standardized Screening Index, (SCI) Standardized Case-Mix Index, (SWI) Standardized Work-Up Index, (STI) Standardized Treatment Index, (OPE) Overall Performance Evaluation, (ROPI) Relative Overall Performance Index.

Source: New malignant breast cancer cases from Surveillance, Epidemiology, and End Results (SEER) Program (http://www.seer.cancer.gov) SEER*Stat.

Database: Incidence - SEER 17-Nov 2010 Sub (1973–2008 varying).

### Relative survival as outcome

If actually observed and expected chances of survival probabilities are related to each other, relative survival rates are obtained [14]. The former originate from empirical data (e.g. registers). But in contrast, the latter can be calculated from so-called “prospective probability of death” from period or cohort life tables stratified according to age, gender, calendar year and occasionally also ethnicity [15,16]. These life tables are available from the Federal Agency for Statistics or the Human Mortality Database (HMD). Different methods for the handling of censored cases [17,18] or various observation periods [19-21] are possible. Non-parametric methods to derive relative survival rates are sufficient in this case and can be easily estimated using freely accessible software-solutions (e.g. [22]).

### Standardized indices

All indices have the principle of construction in common. Time and factually-fixed reference objects define the comparative scale (here USA, 2003). This, so called reference object is the best possible expected result (expected, Exp*). All other benchmark objects (observed, Obs) must be compared to this reference scale. Hence, all indices are defined as: index = Obs/Exp*. From a cross-sectional analysis perspective, the benchmark objects (Obs) belong to the same time interval as the reference object (Exp*). From a longitudinal analysis perspective, however, the benchmark objects can originate from different time intervals (Obs_t), while the reference object is fixed in time. To simplify the matter, the time index t is omitted in the following.

### Extended standardized screening index (eSSI)

The central idea of the eSSI is focused on the relative proportion of ill persons per disease stage (N_i/N) weighted by the stage number itself. The products are then summed up for the benchmark object. The sum is then put into relation with the sum of the reference object which characterizes the standardization-process. The index is defined as:

$$\begin{array}{l} \mathrm{eSSI}=O/E*\\ \;={\left[\sum \left(N\_\_\_i/N\right)\times i\right]}_{O}/{\left[\left(N\_\_\_i/N\right)\times i\right]}_{E*}. \end{array}$$

### Standardized case-Mix index (SCI)

The central idea of the SCI is to multiply the absolute number of ill persons per disease stage (N_i) with the relative survival rate (RSR_i). The products are then summed up and divided by the total number of ill patients. The index is standardized by comparing benchmark to reference objects and is defined as:

$$\begin{array}{l} \mathrm{SCI}=O/E*\\ \;={\left(\left(\sum N\_\_\_i\times \mathrm{RSR}\_\_\_i\right)/N\right)}_{O}/{\left(\left(\sum N\_\_\_i\times \mathrm{RSR}\_\_\_i\right)/N\right)}_{E*}. \end{array}$$

### Standardized work-Up index (SWI)

The central idea of the SWI is to relate the relative survival rate per stage (RSR_i) of a benchmark object (Obs) with the RSR_i of the reference object (E*). The resulting proportions are then summarized. Finally, to get an idea of the average relative survival rate across the stages, the sum is divided by the number of represented disease stages i. The same is true for the reference object. This index is defined as:

$$\mathrm{SWI}=O/E*=\left({\sum \left(\mathrm{RSR}\_\_\_i\right)}_{O}/{\left(\mathrm{RSR}\_\_\_i\right)}_{E*}\right)/N\left(i\right).$$

### Standardized treatment index (STI)

The central idea of the STI is to set the overall relative survival rate (RSR) of benchmark objects (O) and reference objects (E*) in relation to each other. However, since benchmark objects and reference objects can differ in their stage distribution, the SCI is needed as an indicator of risk adjustment. The index is defined as:

$$\mathrm{STI}=O/E*=\left(\mathrm{RSR}\_\_\_\mathrm{Obs}/\mathrm{RSR}\_\_\_\mathrm{Exp}*\right)\times 1/\mathrm{SCI}\_\_\_\mathrm{obs}$$

### Composite measures

According to Beatty et al. [6,7] SCI, SWI, and STI may be summarized to an overall performance evaluation (OPE): OPE= SCI × SWI × STI. As an alternative the relative overall performance index (ROPI) is suggested. The ROPI is defined as:

ROPI=1/eSSI×SWI×STI.

### Example of use

The data of new malignant breast cancer patients from the Surveillance Epidemiology and End Results (SEER17-Nov2010) [12] and the Norwegian cancer register [13] were used. The national SEER17-values from 1999–2003 served as reference objects in the cross-sectional analyses. Thus, these analyses were restricted to the period from 1999–2003 and the seventeen SEER-registers which define the benchmark objects. In the longitudinal analyses, the national SEER17-values from the last available year 2003 served as reference object. The national SEER17 data from 1990–2003 as well as the Norwegian data from the intervals 1969–73, 1974–78, 1979–1983, 1984–88, 1989–93, 1994–98, and 1999–03 [13] served as benchmark objects. The relative 5-year survival rate was calculated according to the Ederer II-method. The examples are available within the additional files (Additional file 1: Example1, Additional file 2: Example2) and can be downloaded from the project-homepage (http://sourceforge.net/projects/benchrelsurv/).

## Results

In the cross-sectional and longitudinal application examples, the reference object was also included as benchmark object. This leads to a special case because benchmark object and reference object are equal. Therefore, the corresponding indices are of value one in cross-sectional analysis. In longitudinal analyses, it leads to index values of one in the chosen reference year (here 2003). This logic allows for all the other benchmark objects that a health service quality gap – or advance – is identified by index values smaller or larger than one. If, for example, eSSI>1 is valid, more patients will be treated at a *later* time point in the benchmark object than in the reference object. If eSSI<1 is valid, more people will be treated at an *earlier* time point than in the reference object. The latter result might be interpreted as a more effective early detection and screening program than in the reference object.

In analogy, for SCI>1, the survival conditions in the benchmark objects will adapt to the normal population’s *faster* than in the reference object. If SCI<1 is measured, the survival conditions in the benchmark object will adapt more *slowly* to the conditions of the population than in the reference object. The latter result might be interpreted as a less effective early detection and screening program than in the reference object.

If SWI>1 is true, then the resumed average survival rates across the stages will be *higher* in the benchmark object than in the reference object. If SWI<1 is true, then the average survival rates across the stages will be *lower* in the benchmark objects than in the reference object. The latter result might be interpreted as a less effective clinical work-up than in the reference object.

If STI>1 is true, then the stage adjusted overall survival rates in the benchmark object will be *higher* than in the reference object. If STI<1 is true, then the stage adjusted overall survival rates in the benchmark object are *lower* than in the reference object. The latter result might be interpreted as a less effective overall treatment than in the reference object.

**Figure 1:**
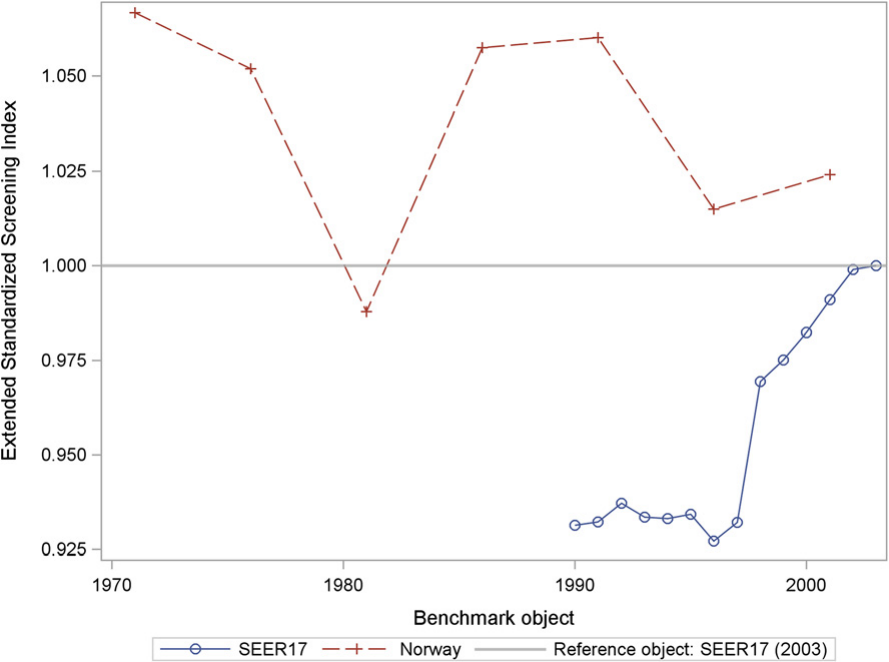
Extended Standardized Screening Index (eSSI) for new malignant breast cancer cases from SEER-17 (1990–2003), Norway (1969–2003) and SEER-17 (2003) as a reference object.

**Figure 2:**
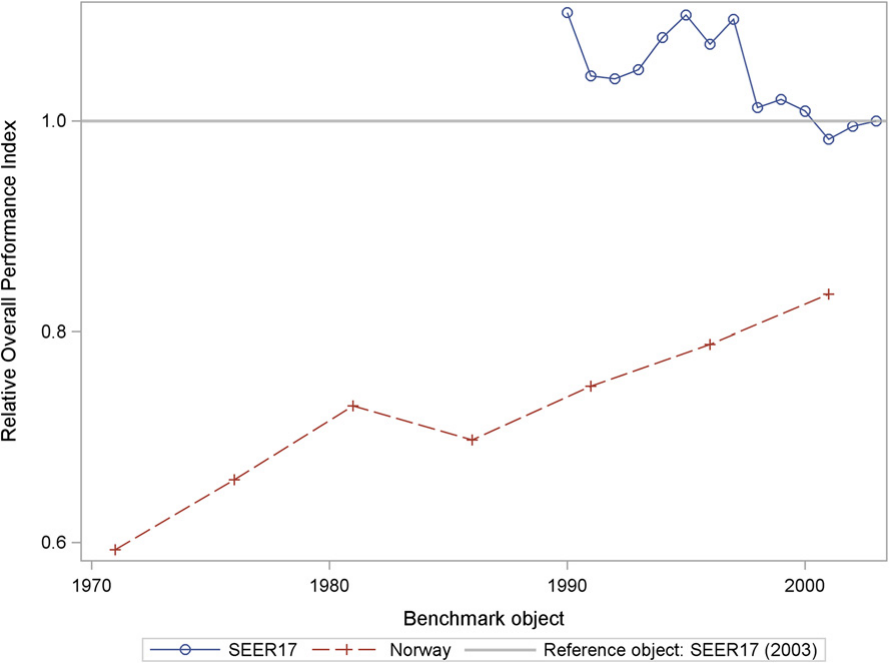
Relative Overall Performance Index (ROPI) of new malignant breast cancer cases from SEER 17 (1990–2003), Norway (1969–2003) and SEER-17 (2003) as a reference object.

If OPE or ROPI>1 is valid, then patients in the benchmark object will have *earlier* treatment with *higher* relative survival rates on average than in the reference object. If OPE or ROPI<1 is true, then patients in the benchmark object will have *later* treatment with *lower* relative survival rates on average than in the reference object. Table 2 shows the cross-sectional results. Examples of longitudinal results for the eSSI and ROPI are depicted in the Figures 1 and 2. The interpretation follows the general instructions.

## Discussion

The existing, updated and extended standardized indices form an additional tool for the evaluation of health care services and quality. Reference objects can be defined and compared to benchmark objects such as countries, states, and districts. The indices offer a cross-sectional and longitudinal perspective on benchmark objects. Especially the latter offer the opportunity to demonstrate the relationship between disease stage and chance of survival during the course of time.

### Comparison with other benchmarking-algorithms

The performed cross sectional analysis detects the already observed high variability between urban-metropolitan areas and rural regions which have led to controversial discussions [23,24]. The longitudinal analyses, however, may show the growing influence of comprehensive early detection and screening-programs and -methods, guidelines as well as increasing utilization and participation rates that may lead to more favorable surrogate parameters such as absolute or relative stage distribution. In this respect the approach is very similar to purely descriptive benchmark projects [2,3,25-28], which also have to interpret obtained results within country- and time-specific conditions. From this perspective both approaches (presented benchmark, descriptives) are somewhat complementary because the latter may provide insights in explicitly measured and process-related quality indicators which are based on medical decisions. In exchange, the formulated benchmark algorithms reject the use of criteria-based approaches to estimate theoretically expected cases [1,5,29] in the indices’ denominator. Therefore, the here presented algorithms cannot inform about insufficient respectively inappropriate health care. Furthermore, the proposed approach cannot revise short-term outcomes such as morbidity or 30-day in-house mortality [4,30], because the chosen outcome parameters of relative survival are bound to annually presented life tables. These may also be calculated for shorter time intervals. In this case the estimation of country-specific or regional life tables is recommended [31] for differentiation purposes. Finally, it should be stressed that the developed approach extends study methods that are aimed at comparing country-based relative 5-year survival rates [32-35], because information of absolute and relative stage distribution is linked to the corresponding survival rates.

### Conceptualization and interpretation

The conceptualization and epidemiological interpretation or health policy conclusions should be drawn in the country and time-specific context. Therefore, the interpretation of the data examples remains restricted here to a.) great variations (cross-sectional) between benchmark objects and b.) convergence tendencies between the US and Norway over the course of time.

One contributing factor for this result is measured by the eSSI and SCI which are both conceptualized on the premise that the more effective early detection and screening programs, the more the distribution of cases will be skewed toward the earlier stages of disease. This characteristic reflects common knowledge regarding the coherence between early detection programs and shifts of stages [36-38] even if screening methods may be discussed as somewhat controversial in certain age groups [39]. Also the conceptualization of the SWI captures this stage migration effect directly. The SWI is based upon the premise that the more critical the work-up, the more upstaging will occur and the better the survival at each stage. However, unless the stage migration alters the treatments administered, it will not impact the overall survival, only the survival at each individual stage. The STI is based upon the premise that the better the overall survival corrected for the case-mix, the more effective the treatment being administered. The utilization of each of these indices as a benchmark provides a means of identifying specific areas of program strength and weakness. The combination of these indices to create ROPI provides a benchmark for assessment of overall program quality.

### Conceptional pitfalls

The suggested method allows the comparison of countries, states, and districts on a longitudinal scale. This perspective offers a high information grade in international comparisons. However, this means that an especially high data quality is necessary that must fulfill the minimum requirements of representative, accurate, complete, and comparable data [40,41]. Aside from these formal requirements, some important statistical details must be regarded which are related to stage distributions and survival rates.

The comparison of the stage distribution may be distorted by the stage migration or the so called “Will-Rogers-Phenomenon“[42-44]. According to this phenomenon slow growing, “quiet”, and not apparently discernible disease symptoms such as metastases are discovered earlier due to increasingly powerful imaging procedures (diagnostic imaging). This means these cases are no longer classified as early disease stages (0-II), but as later ones (III-IV). Thus, the chance of survival increases in the early disease stages, because fewer patients with metastases and unfavorable prognoses are included. However, the chance of survival also increases in the later stages, because patients with metastases that are not apparent are detected earlier. Due to this effect also known as stage migration distorted stage-specific chances of survival result. The overall chance of survival, however, is not affected by this phenomenon [42].

On the contrary, comparison of SWI and STI facilitates a greater understanding of the contribution of this stage migration effect to the overall outcome. For example, if the stage-specific survival information (SWI) is approximately 1.0 and the overall survival information (STI) is substantially greater than 1.0, the improved overall outcome adjusted for the stage mix (SCI) appears to be primarily a treatment effect. On the other hand, if the SWI is substantially greater than 1.0 but the STI is approximately 1.0, there is a stage migration occurring that does not appear to have a major impact on administered treatments.

Alongside, the comparison of survival conditions in time may also be distorted by the so-called lead-time bias or zero-time shift [42,45]. Accordingly, screening-tests and diagnostic procedures can identify a disease even before the patient develops symptoms. This effect leads to increasing survival times without actually leading to prolongation of life, if the health care effectiveness remains constant.

### Strengths

Compared to the original indices according to Beatty et al. [6,7] an extended screening-index (eSSI) is included, which is standardized in the same logic as all the other indices and which is expressed as a reciprocal. Therefore a positive association between the eSS-Index and the outcome-indicator is established. The latter has been redefined by substituting the breast cancer-specific fatality rates by relative survival rates. This approach has several advantages:

•Overall more patients can be included in the analyses because survival of all patients is of concern; regardless of the cause of death. This leads to a higher statistical power.

•Other causes of death respective frequently reported misleading information [46,47] do not have to be modeled in a competing risk model following Gooley [8].

•Background mortality of the population can be considered in the model.

•The calculation of the non-parametrically estimated relative survival rates does not require distribution assumptions. They can be estimated with existing software solutions appropriately.

•One step forward, relative survival rates may be standardized by variables such as age, gender, ethnicity etc. limited only by the parameters available in life tables.

•Benchmark-algorithms and the outcome-indicator relative survival may be extended to any other disease as long as a classification in subsequent disease phases is possible.

The outcome-indicator relative survival can also be substituted by probabilities. For example, logistic regressions can be calculated to quantify readmission probabilities after inpatient treatment, if the corresponding benchmark parameter exists. Correspondingly, disease phases can be substituted by information on disease severity (e.g. Charlson-score, Elixhauser-index [48,49]) as long as they have an ordinal order.

### Weakness

The proposed eSS-Index serves as a level-parameter (“intercept”) which defines the general premise of the benchmark respective reference object. Its weakness is based on the arbitrary weights provided by the stage numbers which devaluate the earliest disease identified (stage 0) and emphasize the “not known” (stage 5, see Table 1). This arbitrariness might decrease the clinical value of both, the eSSI and ROPI. However, eSSI informs about stage distributions without any survival information and obtains the logical consistency of ROPI.

The conceptual pitfalls of the Will-Roger phenomenon, stage migration and lead-time bias have already been mentioned (see above). The former is explored by comparing SWI- and STI, but lead-time bias would distort that assessment and cannot be distinguished from an apparent increase in the incidence of the disease. In addition, it is highly recommended from a methodological point of view to estimate outcome-parameters using the same method but with different regional life tables. Therefore, population-based data is a crucial prerequisite, i.e. the catchment area of integrated networks or new organization forms in general should be clearly defined by their landmarks in order to obtain crucial demographic information from local or regional statistical authorities. In addition, if possible and if these cannot be avoided, benchmark objects should have the same structural breaks in their nomenclature of disease phase (e.g. AJCC, UICC). For example, the UICC Tumor-Node-Metastasis version 5 (1997–2001) was valid until version 6 (2002–2008) and version 7 (since 2009) became effective. The use of the same nomenclature should be assured for reference and benchmark objects. But from a practice point of view, this is difficult to achieve due to time lags in implementation and documentation. Furthermore a fair benchmark has to be assured which means that a comparison between “equivalent” comparison objects should be achieved. Thus, homogenous benchmark objects in terms of “peer-groups” should be identified [50]. This is recommended since most health care systems have evolved historically and, thus, infrastructure characteristics and innovations can be implemented 1:1 from one country, region or district to another under certain restrictions only [51,52]. However, the identification of “peer groups” can either be based on content-related considerations (e.g. countries with national health care services), on statistically chosen disease-specific parameters (e.g. distribution of risk, prognosis, and predictive factors) or both [9,53]. Overall incidence-based factors must be differentiated from patient-, disease-, and health care system-centered factors [45] which are responsible for statistical distortion associated with survival analyses.

### Perspectives

Benchmark-algorithms that compare countries, states, and districts are highly complex and require great attention to research details [40,54]. To be able to take these into consideration, high quality data is necessary [9,53,55]. But high data quality in terms of accuracy and completeness are hard to achieve. In case of the SEER register for example, some concerns have been documented [56] which should be thoroughly considered when results are interpreted for health care decision making. This approach is even more recommended instead of the growing number of health care providers who seek to leave the data gathering process due to cost reductions and missing benefits [57]. However, it is these data that form the basis to achieve a higher transparency of efficiency and health care quality which became a crucial competition parameter in a growing health care industry. Therefore it is crucial for the next step in quality assurance to demonstrate how these data may achieve clues of evidence for further improvements. The algorithms proposed here may serve as first identifier of infrastructural differences in screening programs and compare these with alternated consequences for the clinical work-up in countries, states and districts. However from a methodical point of view, the development of benchmark algorithms is not complete. Corresponding tests are missing to generate p-values for which distribution assumptions are requested. If and under what circumstances certain distributions are given, will be the task of future developments. Finally, the clinical meaning and interpretation of index differences between reference- and benchmark-objects has to be explored in future applications.

## Conclusions

To measure international, national, and regional health care quality, the suggested algorithms and freely accessible SAS-macros BenchRelSurv and BenchRelSurvPlot offer an additional tool to evaluate screening programs, the clinical work-up and effectiveness in general. An effectiveness comparison is sought that links the earliest possible time point of a progressive disease with the time point of an absorbing result after the onset of the primary disease considering the background mortality (relative survival). This is especially relevant for diseases (e.g. breast cancer) where the etiology and disease causes remain unclear. However, this concept can also be transferred to preventable diseases or avoidable mortalities which have clearly defined disease courses (e.g. cardiovascular disease, diabetes mellitus II) and which are clearly avoidable by (behavioral) interventions. The software allows the identification of performance measurement in relation to comparative regions. It offers a first step towards an in depth research analysis.

## Availability and requirements

Project name: Benchmarking relative survival (BenchRelSurv, BenchRelSurvPlot)

Project home page: http://sourceforge.net/projects/benchrelsurv/ delivers files and examples as well as Technical Reports in German and English

Operating system(s): Platform dependency of SAS 9.2 or higher

Programming language: SAS 9.2 and higher

Other requirements: None

License: None

Any restrictions to use by non-academics: SAS 9.2 license or higher

## Competing interests

Authors declare no competing interest.

## Authors’ contributions

COJ contributed substantially to the conception, acquisition of data, design and programming of the source code, analysis and interpretation of data, drafting the manuscript, and gave final approval. IR was involved in the design, programming, and validation of the source code and revised and approved the final draft. USA was involved in the conception, analysis, and interpretation of data, revised critically for important intellectual content, and gave approval for the final draft. All authors read and approved the final manuscript.

## Pre-publication history

The pre-publication history for this paper can be accessed here:

http://www.biomedcentral.com/1471-2458/13/34/prepub

## Supplementary Material

Additional file 1 Cross-sectional benchmark for new malignant breast cancer cases from SEER-17 (1999–2003) registers with BenchRelSurv and BenchRelSurvPlot. Click here for file

Additional file 2 Longitudinal benchmark for new malignant breast cancer cases from SEER-17 (1990–2003), Norway (1969–2003) with BenchRelSurv and BenchRelSurvPlot. Click here for file
